# Individual phenotypic variation reduces interaction strengths in a consumer–resource system

**DOI:** 10.1002/ece3.1212

**Published:** 2014-09-05

**Authors:** Jean P Gibert, Chad E Brassil

**Affiliations:** School of Biological Sciences, University of Nebraska - LincolnManter Hall, Lincoln, Nebraska, 68588-0118

**Keywords:** Interaction strengths, intraspecific variation, invasion, species persistence, stability

## Abstract

Natural populations often show variation in traits that can affect the strength of interspecific interactions. Interaction strengths in turn influence the fate of pairwise interacting populations and the stability of food webs. Understanding the mechanisms relating individual phenotypic variation to interaction strengths is thus central to assess how trait variation affects population and community dynamics. We incorporated nonheritable variation in attack rates and handling times into a classical consumer–resource model to investigate how variation may alter interaction strengths, population dynamics, species persistence, and invasiveness. We found that individual variation influences species persistence through its effect on interaction strengths. In many scenarios, interaction strengths decrease with variation, which in turn affects species coexistence and stability. Because environmental change alters the direction and strength of selection acting upon phenotypic traits, our results have implications for species coexistence in a context of habitat fragmentation, climate change, and the arrival of exotic species to native ecosystems.

## Introduction

Individuals of the same population often show extensive variation in morphology (Bolnick et al. 2003), phenology (Dupont et al. 2011), behavior (e.g., Tinker et al. 2008), and resource utilization (e.g., Estes et al. 2003). This variation can arise from underlying genetic diversity (Lynch and Walsh 1998), or be plastic and result from environmental variability and genotype-by-environment interactions (Fordyce 2006). The importance of genetic and phenotypic variation within populations has long been recognized by evolutionary biology, as heritable individual variation constitutes the raw material upon which natural selection can act (Dobzhansky 1937). Despite a long tradition of considering variation in ontogenetic stages and size within populations, ecological theory has largely overlooked individual variation in its broader sense (Lomnicki 1988). Populations are generally treated as collections of homogeneous individuals and mean demographic parameters, such as mortality or attack rates, are generally used to study population and community dynamics (Sherratt and MacDougall 1995). However, mean demographic rates can be misleading (Inouye 2005), as individual variation may affect demographic parameters and ecological attributes in multiple ways (Bolnick et al. 2011; Pettorelli et al. 2011).

Extensive individual phenotypic and dietary variation has been described for several organisms such as carnivorous marine mammals (e.g., Harcourt 1993), pollinating insects (Dupont et al. 2011), marine, and fresh water fish (e.g., Vander Zanden et al. 2000), as well as several bird species (e.g., Golet et al. 2000). However, only a handful of studies assessed the effect of individual variation upon demographic or ecological traits (e.g., Lloyd-Smith et al. 2005; Melbourne and Hastings 2008). For example, individual variation in resource utilization among southern sea otters (*Enhydra lutris nereis*) structures population-level consumer–resource networks in predictable ways (Tinker et al. 2012). This dietary variation leads to differences in energy intake among individuals, as well as to differences in individual mortality rates through differential pathogen exposure (Tinker et al. 2008; Johnson et al. 2009). Another study showed that the mean reproductive rate of sockeye salmons (*Oncorhyncus nerka*) increases over long time spans with increasing individual variation in life-history traits through a portfolio effect (Greene et al. 2010). Finally, coexistence could theoretically increase with increasing levels of individual variation in attack rates in apparent competition systems with heritable trait variation (Schreiber et al. 2011), and stability could be enhanced whenever behavioral variation is included in consumer–resource systems (Okuyama 2008). Together, these results suggest that the consequences of individual phenotypic variation for population and community dynamics can be important.

Populations embedded in large, complex networks of interacting species such as food webs, often show variation in antipredator defense (Duffy 2010), competitive ability (Lankau and Strauss 2007), or resource utilization (e.g., Estes et al. 2003), all of which can affect interspecific interactions (Pettorelli et al. 2011). The strength of these interactions influences the fate of pairwise interacting populations (e.g., Wootton and Emmerson 2005) and food-web stability (e.g., May 1972; Allesina and Tang 2012). Thus, any factor influencing interaction strengths could affect species persistence and stability in consumer–resource systems. To fully understand food-web stability as well as population and community dynamics, we need to assess the effects of individual variation on ecological attributes that determine the strength of consumer–resource interactions.

**Figure 1:**
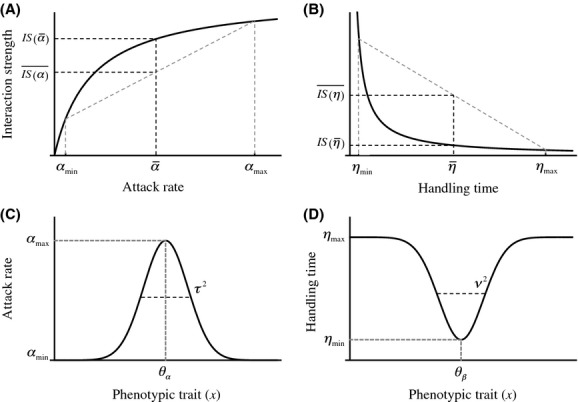
(A), (B); Plots of the magnitude of the interaction strength against attack rate and handling time. Gray dashed curves represent mean interaction strength, not considering individual variation in attack rates or handling times. Solid curves represent interaction strengths considering variation in mean attack rate and handling times. If variation in attack rate only is considered, mean interaction strengths (dashed) are expected to be smaller than actual interaction strengths. If variation in handling time only is considered, mean interaction strengths (dashed) are expected to be greater actual interaction strengths. (C), (D); Plots of attack and handling time against a given quantitative phenotypic trait, where *θ*_*α*_ and *θ*_*η*_ are the optimal trait values for attack rate and handling time, respectively.

Bolnick et al. (2011) identified several mechanisms through which individual variation could affect interaction strengths, including adaptive and stochastic eco-evolutionary feedbacks, increased food-web connectivity, portfolio effects, phenotypic subsidy, and Jensen’s inequality. The latter, a mathematical rule, implies that mean interaction strengths can differ from the interaction strength of the mean individual of the population whenever the variable trait or attribute has purely concave up or down effects on interaction strengths (Jensen 1906; Ruel and Ayres 1999), like attack rates or handling times do (Bolnick et al. 2011). Typically, interaction strengths have been assumed to be functions of mean attack rates and handling times, but, because of Jensen’s inequality, this approach may miss crucial aspects of population and community dynamics. For example, individual variation in attack rates may decrease mean interaction strengths, while individual variation in handling times may increase mean interaction strengths (Fig. 1A and B, Bolnick et al. 2011). However, because attack rate and handling times are not independent from each other (DeLong and Vasseur 2012), it is important to understand what would happen when there is individual variation in both ecological attributes at the same time, as it may occur in a natural system.

In this study, we address how nonheritable individual variation in attack rates and handling times affect interaction strengths within consumer–resource interactions and how this in turn can affect consumer–resource dynamics, species coexistence and overall stability. To do so, we included individual variation in traits controlling attack rate and handling time in classic consumer–resource models to assess how different levels of individual variation might affect ecological dynamics, species persistence and stability in simple consumer–resource models. By doing so, this study answers the following questions: What is the effect of individual variation on interaction strengths? How does this effect alter ecological dynamics and stability? We found that individual variation in attack rate and handling time can increase species persistence and stability through its effect upon interaction strengths. This has in turn important implications for the conservation of endangered species and the management of exotic ones.

## Materials and Methods

### Interaction strengths in classic consumer–resource models

In a consumer–resource interaction model, consumer populations grow through ingesting a resource, which affects the growth rate of that resource (e.g., Rosenzweig and MacArthur 1963). The rate of change of resource and consumers over time can be modeled as:

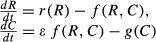
(1)

where *f*(*R*, *C*) and *g*(*C*) are the mortality rates for resource and consumers, respectively, and *r*(*R*) and *εf*(*R*,*C*) are the reproductive rate of resource and consumers, respectively. The functional form of *f*(*R*,*C*) is typically assumed to be the same for both consumers and resources, but its magnitude is scaled in the consumer equation by an efficiency parameter, *ε*, that can take any non-negative real value. May defined interaction strengths (*IS*, from now on) in systems like (eq. 1) as the change in the rate of change of one of the species relative to a small change in the other species’ density. Here, we use May’s definition on a per-capita basis, as advocated by Laska and Wootton (1998), that is, 
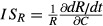
 for resource and 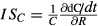 for consumers. Applying this definition to equation (1), we obtain:

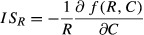
(2)

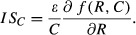
(3)

If we further assume a Holling type II functional response (Holling 1959), where 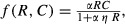 we can get expressions for these interaction strengths that depend on the main parameters controlling the consumer–resource interaction:

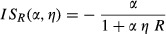
(4)

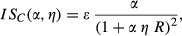
(5)where α denotes the predator’s attack rate and *η* its handling time. Because attack rates and handling times are ecological attributes that depend on phenotypic traits, it is possible to incorporate variation in those traits into equations (4) and (5).

### Incorporating individual variation

In a previous theoretical study, attack rates were assumed to depend on the value, *x*, of a quantitative trait (Schreiber et al. 2011). Here, we assumed that both attack rate and handling time depend on the value of a normally distributed quantitative trait with mean 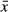 and variance σ^2^. The probability density function of such a trait is

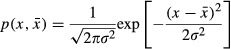
(6)

Following (Schreiber et al. 2011), we assumed the predator’s attack rate, *α*(*x*), to be maximal at a given optimal trait value *x* = *θ*_*α*_, and to then decrease away from that maximum in a Gaussian way:

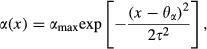
(7)where *α*_max_ is the maximal attack rate and *τ*^2^ determines how steeply the attack rate declines away from *θ*_α_ (Fig. 1C). We further assumed the handling time, *η*(*x*), to be minimal at a given optimal value *x* = *θ*_*η*_, and to increase away from that minimum in a Gaussian way:

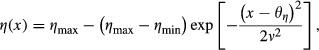
(8)where *η*_max_ and *η*_min_ are the maximal and minimal handling times, respectively, and *ν^2^* determines how steeply the handling time increases away from *θ*_*η*_ (Fig. 1D).

The assumed functional forms for the attack rate and the handling time have been reported for a variety of organisms when body size is considered as the underlying trait of interest (Rall et al. 2012). Our model also assumes that the attack rate and the handling time have inverse functional forms: while attack rate goes down as the trait moves away from the optimum, handling time goes up. The latter is justified by recent empirical work in protists revealing that attack rate and handling time are negatively correlated (DeLong and Vasseur 2012).

We define 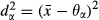 and 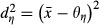, as the squared distance between the mean trait in the population and the optimal value. The optimal value is set by past and existing selective pressures and is the value at which attack rate is maximal and handling time is minimal (referred to as phenotypic mismatch). Phenotypic mismatch can be seen as a measure of how well adapted the predator species is at attacking and handling a particular resource. The larger the mismatch is the smaller the attack rate and the larger the handling time. Phenotypic mismatch has been shown in other traits to affect ecologic interactions and speciation (Raimundo et al. 2014), as well as individual fitness (Anderson et al. 2010). However, it does not need to be the same for both attack rate and handling time, but was assumed to be so for simplicity throughout the main text (but see Appendix S1 and S2 for different assumptions).

To get mean interaction strengths, we thus integrated interaction strengths across the nonlinearity of the functional response and the underlying trait distribution as:

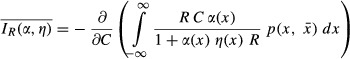
(9)

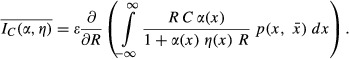
(10)

Using Leibniz integration rule, the derivatives can be passed under the integral sign and equations (9) and (10) can be simplified as:

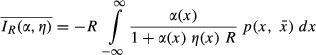
(11)

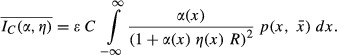
(12)

Equations (11) and (12) depend on individual variation (σ^2^) as well as phenotypic mismatch (*d*^2^) and can be estimated numerically either at equilibrium (when *C* and *R* are constant), or instantaneously (for any given time t).

### General dynamics

To explore the effect of individual variation on consumer–resource interactions and species persistence through interaction strengths, we explored the dynamics of a Rosenzweig–MacArthur consumer–resource model (Rosenzweig and MacArthur 1963). We analyzed the behavior of the model under varying levels of individual variation using:

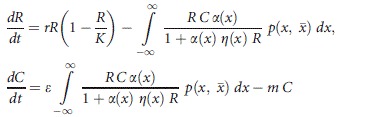
(13)where *K* is the carrying capacity for the resource, *m* is the mortality rate of the consumer and all other parameters are as explained before. Our main objective is to tie the dynamic effect of phenotypic variation on attack rate and handling time through their effect on interaction strengths.

### General questions

In this study, we specifically addressed the following questions: First, does individual variation affect the magnitude of the interaction strength between consumers and resources? We addressed this question by evaluating equations (11) and 12 under increasing levels of individual variation. We also assessed how sensitive interaction strengths were to variation in attack rate and handling time by quantifying their elasticity for varying levels of individual variation (Appendix S3).

Second, if individual variation affects interaction strengths, it can potentially affect population dynamics through the latter. So, would individual variation affect species persistence in a consumer–resource interaction? And, would individual variation affect the stability of consumer–resource interactions? To address these, we derived the conditions for consumer persistence. We also used model equation (13) to assess how individual variation affected the consumer–resource dynamics and found approximate minimal levels of variation needed to achieve stable dynamics. Our approach mimics what is observed in the field (e.g., Matthews et al. 2010), where normally distributed quantitative traits affect the individual use of resources through attack rates and handling times (e.g., Robinson 2000). However, both trait distributions and ecological attributes may not be symmetric in nature; for example, trait distributions may be log-normal (e.g., Gouws et al. 2011) and attack rates may be asymmetric (Vucic-Pestic et al. 2010). We therefore explored three other possible scenarios: (1) trait distributions are asymmetric (Appendix S4), (2) handling time and attack rate are asymmetric functions of the underlying trait *x* (Appendix S5), and (3) both the trait distribution and the functions relating handling time and attack rate to the underlying phenotypic trait are asymmetric (Appendix S6).

## Results

### Interaction strengths

**Figure 2:**
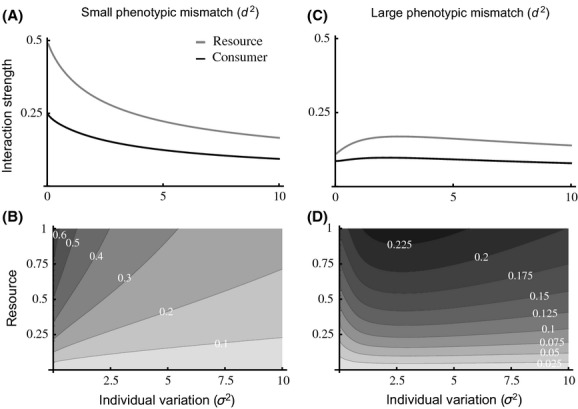
(A), (D); Plots of interaction strength against individual variation (*σ*^2^) for consumers (black) and resources (gray). (B), (D); Contour plots of the interaction strength for varying resource levels against increasing individual variation (*σ*^2^). Small phenotypic mismatch: left column. Large phenotypic mismatch: right column. Parameter values: (A) *α*_max_ = 1, *η*_max_ = 2, *η*_min_ = 1, *ε* = 0.5, *τ* = 1, *ν* = 1, *d*_*α*_ = 0, *d*_*η*_ = 0, *R* = 1; (B) same as in (A) but R varies from 0 to 1; (C) same as in (a) but for *d*_*α*_ = 2, *d*_*η*_ = 2; (D) same as in (C) but R varies from 0 to 1.

When phenotypic mismatch is small (*d*_*α*_ ~ 0 and *d*_*η*_ ~ 0), interaction strengths decay in both consumers and resources with increasing individual variation (Fig. 2A). This is also true under varying resource levels (Fig. 2B). In contrast, if phenotypic mismatch is sufficiently large 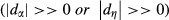, interaction strengths first increase with variation, and then decrease (Fig. 2C), which is also true for varying resource levels (Fig. 2D). These effects seem to increase with resource levels in all cases (Fig. 2B and D). Increasing phenotypic mismatch leads to smaller interaction strengths across all levels of variation (Fig. 2A and C). Our results are robust to changes in the underlying assumptions such as incorporating asymmetric trait distributions (Appendix S4), incorporating asymmetric attack rates and handling times (Appendix S5), or both asymmetric distributions and asymmetric attack rates and handling times (Appendix S6). These results are robust to changes in parameter values (Appendix S1). Notice, however, that asymmetric distributions alone enlarge the range of possible scenarios where interaction strengths decrease with individual variation while the opposite is true for asymmetric attack rate and handling time, regardless of the underlying distribution (Apendices S4, S5, and S6).

### Persistence and stability

For a consumer to be able to persist, the following inequality must hold:

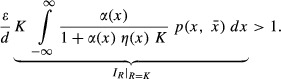
(14)

Notice that the absolute value of the interaction strength experienced by the resource at its carrying capacity (i.e.,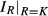) from equation (11) is embedded in equation (14). We know that 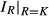 depends on individual variation (σ^2^) such that equation (14) is

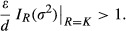
(15)

**Figure 3:**
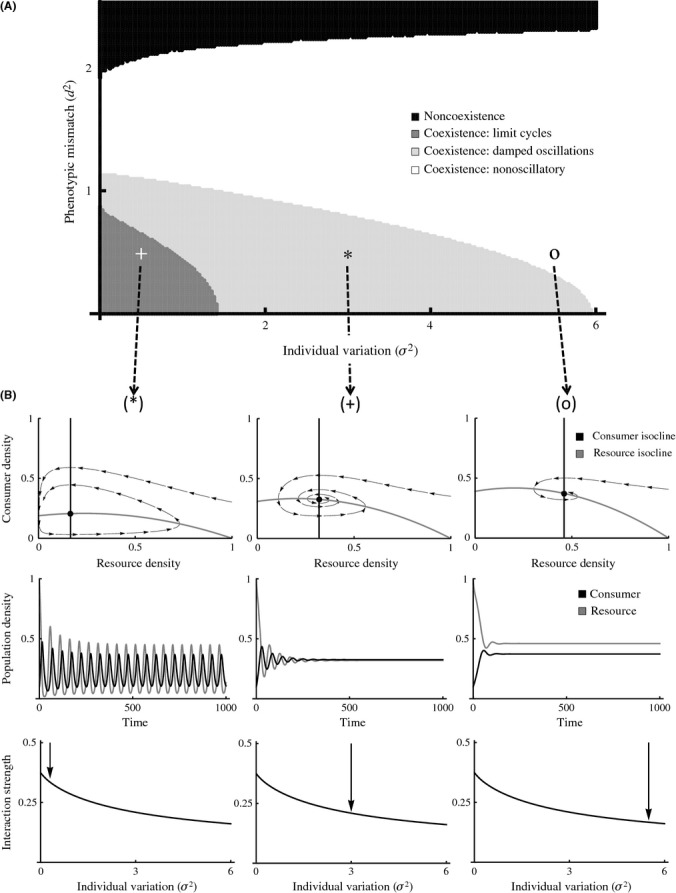
(A) Outcome of the interaction plotted against individual variation and phenotypic mismatch. Consumers can go extinct but the resource survives (black), or both species can coexist (limit cycles in dark gray, damped oscillations light gray, nonoscillatory behavior in white). The asterisk, the cross, and the zero represent combinations of parameters we use as an example of how coexistence, stability, and interaction strengths change with variation. (B) First row: phase diagrams where the equilibrium occurs at the intersection the two isoclines (black: consumers, gray: resource, black dot: equilibrium). Arrows represent one possible trajectory of the system. Second row: dynamics for consumers (black) and resources (gray) through time. Third row: mean interaction strength in the system for both interacting species against individual variation. Parameter values: (a) *r* = 0.3, *α*_max_ = 2, *η*_max_ = 2, *η*_min_ = 1, *ε* = 0.5, *τ* = 1, *ν* = 1, *K* = 1, *β* = 0.1, *d*_*α*_ = *d*_*η*_ = 0.5 and σ^2^ = 0.3 (asterisk); *d*_*α*_ = *d*_*η*_ = 0.5 and σ^2^ = 3 (cross) and *d*_*α*_ = *d*_*η*_ = 0.5 and σ^2^ = 5.5 (zero).

Hence, if phenotypic mismatch is small (*d*_*α*_ ~ 0 and *d*_*η*_ ~ 0), the consumer is less likely to persist since 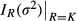 decreases monotonically with individual variation and equation (16) becomes less likely to hold (Fig. 3A). When phenotypic mismatch is large 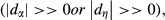 the likelihood of consumer persistence gets larger at first and then decreases (Fig. 3B), following the effect of individual variation on interaction strengths (Fig. 2). The larger the phenotypic mismatch, however, the less likely the persistence criteria will be met, as the interaction strength becomes consistently smaller with variation (Fig. 2).

Increasing phenotypic mismatch decreases consumer persistence regardless of individual variation (Fig. 3A). Increasing levels of variation can counter this effect by rescuing consumers from extinction under some conditions, and by stabilizing consumer–resource interactions (Fig. 3A). For a given level of phenotypic mismatch, an increase in individual variation can be accompanied by a change in persistence; from noncoexistence to coexistence, and a change in dynamics; from limit cycles to oscillatory dynamics to nonoscillatory dynamics Fig. 3A and Fig. 3B, first, and second rows). Increasing individual variation not only increases stability, but decreases interaction strengths concomitantly (Fig. 3B, third row). Both phenotypic mismatch and individual variation affect species coexistence through altering resource and consumer isoclines: the consumer isocline shifts to the right while the resource isocline moves up with increasing levels of individual variation (Fig. 3B first row). Nevertheless, extremely large values of individual variation can drive consumers to extinction, as they are no longer able to ingest resource at a high enough rate (Appendix S7, also eq. 15). Although Jensen’s inequality predicts opposite effects of variation in attack rate and handling time when considered independently (Fig. 1A and B), the effects of individual variation upon the consumer–resource dynamics seem to be mainly driven by variation in the attack rate (Appendix S3).

These numerical results are in accordance with our analytic predictions, where the condition for stability can be approximated as:

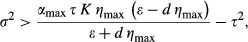
(16)whenever variation on attack rates has a larger effect on dynamics than that of handling time, phenotypic mismatch is small (*d*_*α*_ ~ 0 and *d*_*η*_ ~ 0), and variation is small enough (Appendix S8 for the derivation). Here, *d* stands for the consumer death rate. In this case, the system is stable if individual variation is larger than a certain quantity that increases with the maximal attack rate (*α*_max_), the carrying capacity (*K*) and the digestive efficiency (*ε*). Notice that equation (16) resembles the CV rule of Hassell et al. (1991), where the ccoefficient of variation squared needs to be larger than 1 for a spatially variable consumer–resource parasitoid interaction to be stable.

Combined, these results suggest that the effect of variation in attack rates is dominant over that of handling times (Appendix S3), which leads to a reduction in interaction strengths (Fig. 2), and an increase in coexistence and stability (Fig. 3), unless variation is too large (eq. 15, Fig. 3).

## Discussion

Individual variation in demographic parameters is pervasive in most systems (Bolnick et al. 2003), but only a handful OF studies have addressed the potential effects of this variation on population dynamics and species persistence (Okuyama 2008) or eco-evolutionary dynamics (Schreiber et al. 2011; Vasseur et al. 2011). Here, we show that nonheritable individual variation may drive ecological consumer–resource interactions through its effect on interaction strengths, as suggested by recent empirical studies (Agashe 2009; Jones and Post 2013). This effect may vary with the environment, and should be different for species with different levels of phenotypic mismatch, ultimately caused by past and existing levels of stabilizing selection. In what follows, we propose testable predictions with respect to a possible trade-off between persistence and biological invasiveness mediated by phenotypic variation. Finally, we show that the effect of individual variation through Jensen’s inequality may strongly depend on assumptions regarding the functional form of ecological attributes, which underlines the need for more accurate estimates of trait and ecological attribute distributions using empirical and experimental approaches.

### Interaction strengths, selection, and whole community effects

Although individual variation can increase species persistence in the eco-evolutionary dynamics of an apparent competition system (Schreiber et al. 2011), the mechanisms through which this happens are unclear. Classical models of consumer–resource interactions suggest that larger interaction strengths destabilize equilibrium densities, and bring species closer to extinction thresholds, potentially leading to species extinction (Rosenzweig and MacArthur 1963). Our results are consistent with these classic studies, and by showing how individual variation can reduce interaction strengths, we provide a mechanistic explanation as to why interacting species with larger levels of variation seem to persist more than those with smaller levels of variation (Newman and Pilson 1997; Imura et al. 2003).

However, our results also suggest that the effect of individual variation on interaction strengths depends on the levels of phenotypic mismatch between consumers and resources, and these are ultimately controlled by existing and past selective pressures (e.g., Fellowes et al. 1998; Nuismer et al. 2010). Small phenotypic mismatch can lead to large interaction strengths when variation is small, and can result from strong stabilizing selection. In contrast, large phenotypic mismatch reduces interaction strengths AND can result from weak stabilizing selection, a trade-off with another trait, or a recent environmental shift leading to maladaptation. Also, because constant environments can impose strong stabilizing selection and fluctuating environments can impose weak stabilizing selection (Gavrilets and Hastings 1994; Zhang and Hill 2005), our results suggest that the effect of individual variation may be environment-dependent.

Our results could have important implications for food-web theory. For example, interaction strengths have also long been known to drive the stability of large, complex networks of interacting species such as food webs (e.g., May 1972; Allesina and Tang 2012). Because individual variation affects interaction strengths, our results suggest that, to fully understand why complex food webs are stable in nature, we may need to take into account individual variation. For example, weak interaction strengths have been suggested to increase overall stability (McCann et al. 1998), and we show here that weak interaction strengths occur with high individual variation or phenotypic mismatch. Hence, stable food webs may be characterized by species with high levels of individual variation and small phenotypic mismatch between consumers and resources, or by a mixture of species with low and high levels of individual variation, provided that phenotypic mismatch is large enough among species. Conversely, unstable food webs may be characterized by species with low levels of individual variation and small phenotypic mismatch. Testing some of these ideas in empirical food webs could strongly advance our understanding of how large complex food webs persist in nature despite their structural instability. Unfortunately, this may not be currently feasible.

### Individual variation and biological invasions

We showed that variation can affect interaction strengths and species persistence. In what follows, we argue that this could have important consequences for the establishment of biological invaders. For small phenotypic mismatch between consumers and resources, interaction strength decreases monotonically with variation (Fig. 2A), which results in an increase in resource persistence but an eventual decrease in consumer persistence (Fig. 3A). The antagonistic effects of individual variation on persistence and stability suggest that invasive consumers able to invade and persist may have intermediate levels of variation whenever phenotypic mismatch is small (Fig. 3A). This prediction can be tested readily in the field and is in line with previous empirical findings on invasive weeds (Genton et al. 2005). Whenever phenotypic mismatch is large, however, the hump-shaped relationship between variation and interaction strengths (Fig. 2C) may lead to successful invasive consumers with either low or high individual variation, both of which have been reported in the field (Estoup et al. 2001; Kolbe et al. 2004, respectively).

Invasive species can enter a new environment with a single or a few individuals and could therefore have low individual variation during the establishment phase (Facon et al. 2006). If phenotypic mismatch is small, the interaction strength with native resource species may be high, and their effect upon native diversity may be devastating. Furthermore, failed attempts to eradicate the invasive species may just reduce the individual variation of the invasive species even more, resulting in stronger interaction strengths and deteriorated native species persistence. If phenotypic mismatch is large, however, even with moderately high levels of variation, interaction strengths could be quite low. In this case, eradication attempts could effectively reduce individual variation even more, resulting in weaker interaction strengths and improved species persistence provided that phenotypic mismatch does not change much over time. Finally, our results strengthen previous findings suggesting that the probability of a successful invasion depends on underlying variation (Jones and Gomulkiewicz 2012) and stress the need for taking individual variation into account in order to devise better management policies regarding invasive species.

### Jensen’s inequality and a plea for empirical estimation of trait variability

Because of Jensen’s inequality it has been previously suggested that attack rates and handling times could have opposite effects on interaction strengths when individual trait variation was taken into account in each attribute independently (Fig. 1A and B this paper, Bolnick et al. 2011). Although variation in the traits controlling the attack rate seems to have more profound effects upon ecological dynamics than in those controlling the handling time, our findings also suggest that these predictions are contingent on the specific functional forms through which attack rate and handling time depend on underlying phenotypic trait variation. Hence, our results emphasize the need for gathering estimates about how ecologically relevant traits distribute in real populations, and assessing the functional form of their effect upon ecological attributes.

One possible way of doing so is to use controlled microcosm experiments of consumer and resource protists (e.g., DeLong and Vasseur 2013), where attack rates and handling times could be directly measured while underlying phenotypic variation is manipulated. These systems are particularly well suited for quantifying entire trait distributions (DeLong 2012) and are thus prime candidates to test some of our ideas. Indeed, previous mesocosm studies assessed the effect of variation in defense traits in algal populations, showing that variation in defense mechanisms could alter biological dynamics (Yoshida et al. 2004). Hence, while difficult, it is not impossible to gather some of this information in fairly complex empirical systems.

## Conclusions

Our results are in accordance with previous theoretical studies that have shown that increased behavioral variation (Okuyama 2008) and variation in the use of space by parasitoids in heterogeneous landscapes (Hassell et al. 1991) are mostly stabilizing. Moreover, we derived conditions for stability that are qualitatively similar to those derived by Hassell and collaborators, which together suggest that there is a minimal threshold of variation below which ecological dynamics become highly unstable. We also note that spatial or environmental heterogeneity, as considered in the work by Hassell et al. (1991), can induce differences in space use among individuals. This variation in space use ought to be regarded as a type of individual phenotypic variation, and we thus argue that these converging results may be due to variation decreasing interaction strengths through Jensen’s inequality.

Other researchers have explored consumer–resource dynamics in the case where there is behavioral variation in foraging rates (Okuyama 2008); however, our approach differs from theirs in several important ways: first, we explicitly modeled variation in underlying quantitative phenotypic traits controlling attack rates and handling times, only making assumptions grounded on biological data; second, we accounted for the potential effects of phenotypic mismatch, or the difference between mean trait in the population and the adaptive peak; and last, we have drawn a mechanistic link between individual variation and population dynamics by exploring its effect on interaction strengths. The latter is the ultimate link to connect pairwise models to whole food-web dynamics and stability (e.g., May 1972; Allesina and Tang 2012).

Overall our study shows that individual variation can affect species persistence and coexistence between consumer and resource through its effect on interaction strengths. Moreover, the effect of individual variation on interaction strengths depends on phenotypic mismatch and thus, on current and past selective pressures. This has important implications for species persistence embedded in food webs or the arrival of invasive species to native ecosystems. Finally, this study underlines the need for accurately estimating the distribution of ecologically relevant phenotypic traits, as well as their functional relationship with ecological attributes, in order to test our predictions of how individual variation affects the ecology and persistence of interacting populations.

## Supporting Information

**Appendix S1.** Parameter values. **Appendix S2.** Attack rate and Handling time. **Appendix S3.** Elasticity for attack rates and handling times. **Appendix S4.** Asymmetric trait distribution. **Appendix S5.** Asymmetric functional forms for the attack rate and the handling time. **Appendix S6.** Asymmetric trait distribution and asymmetric attack rate and handling time. **Appendix S7.** Species persistence. **Appendix S8.** Stability.
