# Supplementary material for: Individual phenotypic variation reduces interaction strengths in a consumer–resource system
Source: Ecol Evol. 2014 Sep 5;4(18):3703–13. doi: 10.1002/ece3.1212 (PMC4224542; doi:10.1002/ece3.1212)
Supplement: Supplementary file 1 — Appendix S1. Parameter values. Appendix S2. Attack rate and Handling time. Appendix S3. Elasticity for attack rates and handling times. Appendix S4. Asymmetric trait distribution. Appendix S5. Asymmetric functional forms for the attack rate and the handling time. Appendix S6. Asymmetric trait distribution and asymmetric attack rate and handling time. Appendix S7. Species persistence. Appendix S8. Stability. [file ece30004-3703-sd1.docx]

ONLINE SUPPORTING INFORMATION:

Individual phenotypic variation reduces interaction strengths in a consumer-resource system

Jean P. Gibert and Chad E. Brassil

INDEX

Appendix 1: Parameter values ..……………………………………………………….. 2 - 4

Appendix 2: Attack rate and Handling time ...………………………………………… 5

Appendix 3: Elasticity for attack rates and handling times …………………………….. 6 - 7

Appendix 4: Asymmetric trait distribution …………………………………………….. 8 - 10

Appendix 5: Asymmetric functional forms for attack rate and handling time ………… 11 - 14

Appendix 6: Asymmetric trait distributions and asymmetric attack rate and handling time………………………………………………………………………………………15 - 16

Appendix 7: Species persistence ……………………………………………………… 17

Appendix 8: Stability ……………………………………………………………………18 - 19

References: ………………………………………………………………………………… 20

APPENDIX 1: Exploring different parameter values

In this section we assess how robust our results are to a change in parameter values. We did so by exploring other possible values for $d_{\alpha}$, $d_{\eta}$, $\tau$ and $\nu$. For changes in $d_{\alpha}$ and $d_{\eta}$ our qualitative results hold, but an increase in $d_{\eta}$ seems to have a less pronounced effect than one in $d_{\alpha}$ (Fig. S1-1; also see Appendix 3). As $\tau$ and$\nu$ increase, the effect of individual variation decreases (Fig. S1-2). This occurs because the attack rate and the handling time become constant, and largely independent of the value of the controlling trait. Small $\tau$ or $\nu$ leads to a large dependency of the attack rate and the handling time upon the underlying trait value, and hence, to an increased effect of individual trait variation (Fig. S1-3).

Fig S1-1: Plots of interaction strength against increasing individual variation (gray: resource, black: consumer). (a) $\alpha$= 1, $\eta_{\max}$= 2, $\eta_{\min}$= 1, $\tau$=3, $\nu$= 1, $d_{\alpha}$= 2, $d_{\eta}$= 0. (b) same as (a) but for $d_{\alpha}$= 0, $d_{\eta}$= 2.

Figure S1-2: Plots of interaction strength against individual variation measured as $\sigma^{2}$. Parameter values: (a) $\alpha$= 1, $\eta_{\max}$= 2, $\eta_{\min}$= 1, $\tau$=3, $\nu$= 1, $d_{\alpha}$= 0, $d_{\eta}$= 0. (b) same as (a) but for $\tau$=1, $\nu$= 3. (c) same as (a) but for $\tau$=3, $\nu$= 3.

Figure S1-3: Plots of interaction strength against individual variation measured as $\sigma^{2}$. Parameter values: $\alpha$= 1, $\eta_{\max}$= 2, $\eta_{\min}$= 1, $\tau$=0.1, $\nu$= 0.1, $d_{\alpha}$= 0, $d_{\eta}$= 0.

APPENDIX 2: Mean attack rate and mean handling time

In what follows we show how the mean attack rate and the mean handling time change with increasing levels of individual variation. While attack rate decreases with individual variation whenever phenotypic mismatch is small, handling time increases (Fig. S2-1a). When phenotypic mismatch is large, however, attack rate increases at first with variation and then decreases, and the opposite is true for handling time (Fig. S2-1b).

Figure S2-1: Plots of how mean attack rate (black) and mean handling time (grey) change with individual variation under small phenotypic mismatch (a) and larger phenotypic mismatch (b). Parameter values: (a) $\alpha_{\max}$= 2, $\eta_{\max}$= 2, $\eta_{\min}$= 1, $\varepsilon$= 0.5, $\tau$=1, $\nu$= 1, $d_{\alpha}=d_{\eta}=0$; (b) same as in (a) but for $d_{\alpha}=d_{\eta}=2$.

APPENDIX 3: Elasticity

The elasticity is a measure of model sensitivity defined as the absolute value of $\partial\log(f)/\partial\log(a)$, where $f$ is the function of interest (interaction strength in this case), and $a$ is the parameter of interest (attack rate or handling time in this case). The larger the elasticity, the more sensitive the function is to a change in the parameter.

The effects of individual variation upon consumer-resource dynamics seem to be mainly driven by variation in the attack rate, as its elasticity is generally larger than that of the of handling time regardless of phenotypic mismatch or individual variation (Fig. S3-1). Although Jensen’s inequality predicts opposite effects of variation in attack rate and handling time when considered independently (Fig. 1a, 1b), interaction strengths incorporating individual variation in both attack rate and handling time simultaneously seem to mainly be affected by variation in attack rate.

Fig S3-1: Plot of the elasticity of the interaction strengths for with respect to the attack rate (black) and the handling time (gray). (a) $\alpha$= 1, $\eta_{\max}$= 2, $\eta_{\min}$= 1, $\tau$=1, $\nu$= 1, $d_{\alpha}$= 0, $d_{\eta}$= 0. (b) same as (a) but for $d_{\alpha}$= 2. (c) same as (a) but for $d_{\eta}$= 2.

APPENDIX 4: asymmetric trait distribution

In the main text we assumed the trait that controls the ecological interaction through its effect on attack rate and handling time to be normally distributed. However, the distribution of some traits is highly asymmetric and skewed (Gouws *et al.* 2011). In this section, we break this assumption by incorporating an asymmetric distribution (log-normal distribution, Fig. S4-1). We show that the effect of individual variation is not largely affected by the choice of the underlying trait distribution but the range of scenarios at which interaction strength decreases with individual variation becomes larger when asymmetry is taken into account.

Here, we assumed both attack rate and handling time to depend on the value of a log-normally distributed trait with location parameter $\bar{x}$ and scale parameter $\sigma^{2}$. Then its density in the population is:

. (1)

Note that as both the location and scale parameter control the shape of the distribution, the variance of the distribution, and hence, individual variation, now depends on both parameters. For simplicity, we focus on the case where only $\sigma^{2}$ varies. We have numerically integrated $\overline{I_{R,L}(\alpha,\eta)}$ and $\overline{I_{C,L}(\alpha,\eta)}$ to find the interaction strength with varying levels of individual variation $\sigma^{2}$ as:

$\overline{I_{R,L}(\alpha,\eta)}=-R \int\frac{\alpha(x)}{1+\alpha(x) \eta(x) R} Lp(x, \bar{x}-\infty\infty\int dx$ (2)

$\overline{I_{C, L}(\alpha,\eta)}=\varepsilon C \int_{-\infty}^{\infty} \frac{\alpha(x)}{\left(1+\alpha(x) \eta(x) R\right)^{2}} Lp(x, \bar{x}) dx$ (3)

We found that the interaction strength has a qualitatively similar behavior with respect to individual variation than in the case with a symmetric distribution. This is, there is a range of scenarios at which the interaction strength decreases monotonically with individual variation, and a range of scenarios at which the interaction strength is maximized by intermediate values of individual variation (see main text). Indeed, there is an optimal amount of individual variation that maximizes interaction strength when trait mismatch is large, if the average trait value in the population is smaller than the selective optimum ($d_{\alpha}<<0$or $d_{\eta}<<0$, Fig S4-2a), and this behavior is also quantitatively comparable to the one obtained with a symmetric trait distribution. The interaction strength still decreases with individual variation whenever trait mismatch is small ($d_{\alpha}\sim0$ and $d_{\eta}\sim0$, Fig S4-2b), but this is also true for cases where the average trait value in the population is larger than the selective optimum ($d_{\alpha}>>0$or $d_{\eta}>>0$, Fig S4-2c). Thus, asymmetric trait distributions can increase the range of scenarios in which interaction strengths decreases with individual variation.

Figure S4-1: Plot of a symmetric distribution (e.g. normal) and an asymmetric distribution

(e.g. log-normal). The log-normal distribution used in the supplementary material mainly differs from the normal distribution used in the main text in that it the former is more skewed than the latter.

Figure S4-2: Plots of interaction strength against individual variation measured as $\sigma^{2}$. Phenotypic mismatch is large (a) and (c), and small in (b). Parameter values: (a) $\alpha$= 1, $\eta_{\max}$= 2, $\eta_{\min}$= 1, $\tau$=1, $\nu$= 1, $d_{\alpha}$= -2, $d_{\eta}$= 0. (b) same as (a) but for $d_{\alpha}$= 0. (c) same as (a) but for $d_{\alpha}$= 2.

APPENDIX 5: asymmetric functional forms for attack rate and handling time

In the main text, we assumed the attack rate and handling time to be non-linear, yet symmetric functional forms of the underlying controlling quantitative phenotypic trait. However, these ecological attributes could be asymmetric, as found in most thermal response curves (Vasseur et al. 2014). The asymmetry of these functional forms generally arise from important physiological or biomechanical constrains (Vucic-Pestic et al. 2010), which need to be taken into account to accurately describe the non-linear relationship between underlying phenotypic traits and the ecological attributes they influence. In this section, we break the assumption of symmetry for the attack rate and the handling time, by incorporating asymmetric functional forms (Fig. S5-1). We found that the asymmetry in attack and handling times can have a quantitative effect in the way individual variation affects interaction strengths, mostly by reducing the range of possible scenarios in which interaction strength decreases monotonically with increasing individual variation.

The now asymmetric predator’s attack rate, $\alpha_{asymm}(x)$, can be assumed to be maximal at a given optimal trait value $x=\theta_{\alpha}$, and to decrease away from that maximum at a different rate depending on the direction. Such a scenario can be modeled by:

$\alpha_{asymm}(x)=\alpha_{\max}-\alpha_{\max}\exp\left[-\frac{\left( \log(x)-\log(\theta_{\alpha}) \right)^{2}}{2\tau^{2}}\right]$, (4)

where $\alpha_{\max}$ is the maximal attack rate (Fig. S5-1a) and the rest of the parameters are as described in the main text. Similarly, the predator’s handling time, $\eta_{asymm}(x)$, is minimal at the given optimal value $x=\theta_{\eta}$, and increases away from that minimum at a different rate depending on the direction like:

$\eta_{asymm}(x)=\left(\eta_{\max}-\eta_{\min}\right)\exp\left[-\frac{\left( \log(x)-\log(\theta_{\eta}) \right)^{2}}{2\nu^{2}}\right]$, (5)

where $\eta_{\max}$ and $\eta_{\min}$ are maximal and minimal handling times respectively (Fig. S5-1b) and the rest of the parameters are as described in the main text. Because of the asymmetry, it is now impossible to derive analytic expressions for the mean (asymmetric) attack rate and handling times, so we have numerically integrated $\overline{I_{R, asymm}(\alpha,\eta)}$ and $\overline{I_{C, asymm}(\alpha,\eta)}$ to find the interaction strength with varying individual variation $\sigma^{2}$ as:

$\overline{I_{R, asymm}(\alpha,\eta)}=-R \int\frac{\alpha_{asymm}(x)}{1+\alpha_{asymm}(x) \eta_{asymm}(x) R} p(x, \bar{x}-\infty\infty\int dx$ (6)

$\overline{I_{C, asymm}(\alpha,\eta)}=\varepsilon C \int_{-\infty}^{\infty} \frac{\alpha_{asymm}(x)}{\left(1+\alpha_{asymm}(x) \eta_{asymm}(x) R\right)^{2}} p(x, \bar{x}) dx$ (7)

Overall, we found that the asymmetry in attack rate and handling time seems to preclude a monotonically decreasing relation of interaction strengths with individual variation. If phenotypic mismatch is large enough and the average trait value in the population is smaller than the selective optimum ($d_{\alpha}<<0$or $d_{\eta}<<0$), both the symmetric and the asymmetric case predict a hump shaped relationship between interaction strengths and individual variation. If phenotypic mismatch is small ($d_{\alpha}\sim0$ and $d_{\eta}\sim0$), interaction seems to only increase with individual variation when asymmetric attack and handling rates are considered, rather than showing a monotonic decrease as with symmetric attack rates and handling times (Fig. S5-2b). Finally, if the average trait value in the population is larger than the selective optimum ($d_{\alpha}>>0$or $d_{\eta}>>0$), both the symmetric and the asymmetric case are congruent.

Figure S5-1: Plots of attack and handling time against a given quantitative phenotypic trait, where $\theta_{\alpha}$ and $\theta_{\eta}$ are the optimal trait values for attack rate and handling time respectively. Note that the ecological attributes are now asymmetric with respect to the trait of interest in contrast to what was assumed in the main text (Fig. 2, main text).

Figure S5-2: Plots of interaction strength against individual variation measured as $\sigma^{2}$. Phenotypic mismatch is large in (a) and (c), and small in (b). Parameter values: (a) $\alpha$= 1, $\eta_{\max}$= 2, $\eta_{\min}$= 1, $\tau$=1, $\nu$= 1, $d_{\alpha}$= -3, $d_{\eta}$= 0. (b) same as (a) but for $d_{\alpha}$= 0. (c) same as (a) but for $d_{\alpha}$= 3.

APPENDIX 6: Asymmetric trait distributions, and asymmetric attack rate and handling time

In this section, we incorporate asymmetric trait distributions as well as asymmetric attack rate and handling times by means of equations (1), (4) and (5) of the supporting information. Because of the asymmetry, it is now impossible to derive analytic expressions for the (asymmetric) attack rate and handling time, so we have numerically integrated $\overline{I_{R, Lasymm}(\alpha,\eta)}$ and $\overline{I_{C, Lasymm}(\alpha,\eta)}$ to find the interaction strength with varying individual variation $\sigma^{2}$ as:

$\overline{I_{R, Lasymm}(\alpha,\eta)}=-R \int\frac{\alpha_{asymm}(x)}{1+\alpha_{asymm}(x) \eta_{asymm}(x) R} Lp(x, \bar{x}-\infty\infty\int dx$ (8)

$\overline{I_{C, Lasymm}(\alpha,\eta)}=\varepsilon C \int_{-\infty}^{\infty} \frac{\alpha_{asymm}(x)}{\left(1+\alpha_{asymm}(x) \eta_{asymm}(x) R\right)^{2}} Lp(x, \bar{x}) dx$ (9)

The results for asymmetric distribution and asymmetric attack rate and handling time are comparable to those found in Appendix S5. Specifically, whenever phenotypic mismatch is large enough and the average trait value in the population is smaller than the selective optimum ($d_{\alpha}<<0$or $d_{\eta}<<0$), the symmetric and the asymmetric cases yield comparable predictions (Fig. S6-1a). Conversely, the interaction strength seems to be maximized by intermediate levels of individual variation whenever phenotypic mismatch is small ($d_{\alpha}\sim0$ and $d_{\eta}\sim0$), but this differs from what is predicted by the symmetric case (Fig. S6-1b). Finally, whenever the average trait value in the population is larger than the selective optimum ($d_{\alpha}>>0$or $d_{\eta}>>0$, Fig. S6-1c), both symmetric and asymmetric cases are congruent. Overall, it seems that asymmetric relationships between the attack rate and the handling time with the underlying controlling quantitative trait precludes interaction strengths to decrease with individual variation, but the opposite is truth whenever only asymmetric distributions are considered.

Figure S6-1: Plots of interaction strength against individual variation measured as $\sigma^{2}$. Phenotypic mismatch is large in (a) and (c), and small in (b). Parameter values: (a) $\alpha$= 1, $\eta_{\max}$= 2, $\eta_{\min}$= 1, $\tau$=1, $\nu$= 1, $d_{\alpha}$= -2, $d_{\eta}$= 0. (b) same as (a) but for $d_{\alpha}$= 0. (c) same as (a) but for $d_{\alpha}$= 5.

APPENDIX S7: Consumer persistence

Large values of individual variation can lead to consumer extinction (Fig S7-1), as suggested by eqn 14 and eqn 15 of the main text.

Figure S7-1: Outcome of the consumer-resource interaction as a function of individual variation ($\sigma^{2}$) and phenotypic mismatch between preys and predators ($d^{2}$). In the black region, consumers go extinct but the resource survives, while in white and grey regions both consumers and resources coexist. Parameter values: $\alpha_{\max}$= 2, $\eta_{\max}$= 2, $\eta_{\min}$= 1, $\varepsilon$= 0.5, $\tau$=1, $\nu$= 1, $d_{\alpha}=d_{\eta}$, K=1, $\beta$= 0.1.

APPENDIX S8:

Here we show that for those values of $\sigma^{2}$ for which coexistence is ensured, the larger $\sigma^{2}$ is, the more stable the system becomes. To do so, we observe that, if $\sigma^{2}$ is very small, then the following equality holds,

, (10)

where:

$=\frac{\alpha_{\max} \tau}{\sqrt{\sigma^{2}+\tau^{2}}} \exp\left[-\frac{d_{\alpha}^{2}}{2 (\sigma^{2}+\tau^{2})}\right]$, (11)

$=\eta_{\max}-\frac{\nu\left(\eta_{\max}-\eta_{\min}\right)}{\sqrt{\sigma^{2}+\nu^{2}}} \exp\left[-\frac{d_{\eta}^{2}}{2 (\sigma^{2}+\nu^{2})}\right]$, (12)

and $d_{\alpha}=\bar{x}-\theta_{\alpha}$ and $d_{\eta}=\bar{x}-\theta_{\eta}$, are the distance between the mean trait in the population and the adaptive optimum (phenotypic mismatch).

Hence, assuming that individual variation is small enough, we can assess local stability of the dynamic system by replacing the functional response defined in the main text (in eqn 13 of the main text, or right side of eq. 10 in appendix) by the functional response evaluated at $\bar{\alpha}(\bar{x}$ and $\bar{\eta}(\bar{x}$, and by then calculating the Jacobian of the system at its equilibrium:

. (13)

The system is stable, if and only if the determinant of $\left.J\right|_{R*,C*}$ is positive but its trace is negative. The latter is true whenever:

and . We can now use (11) of the appendix to obtain:

. (14)

If phenotypic mismatch is small ($d_{\alpha}^{2}\sim0$), we can rearrange the eq. 14 to obtain:

. (15)

Finally, if we further assume that variation in attack rate has a larger effect than that in handling time, as observed in appendix 3, we get eq. 3.3 of the main text:

$\sigma^{2}>\frac{\alpha_{\max} \tau K \eta_{\max} \left(\varepsilon-d \eta_{\max}\right)}{\varepsilon+d \eta_{\max} }-\tau^{2}$. (16)

Eq. 16 implies that for the system to be stable, individual variation needs to be larger than a certain amount. This is supported by our simulations (Fig 3, main text), as increasing variation forces the system through a Hopf bifurcation, from an attractive limit cycle to an attractor node. Although the limit cycle is orbitally stable, the population fluctuations underwent by both interacting species makes the system more likely to lose species due to demographic or environmental variability.

REFERENCES:

Gouws EJ, Gaston KJ, Chown SL. 2011 Intraspecific body size frequency distributions of insects. *PloS ONE*, 6, e16606.

Schreiber SJ, Bürger R, Bolnick DI. 2011 The community effects of phenotypic and genetic variation within a predator population. *Ecology*, 92, 1582–93.

Vasseur DA, Delong JP, Gilbert B, Greig HS, Harley CDG, McCann KS, Savage V, Tunney TD, O’Connor MI. 2014 Increased temperature variation poses a greater risk to species than climate warming Increased temperature variation poses a greater risk to species than climate warming. *Proc. R. Soc. B Biol. Sci.* 281.

Vucic-Pestic O, Rall BC, Kalinkat G, Brose U. 2010 Allometric functional response model: body masses constrain interaction strengths. *J. of Anim. Ecol.*, 79, 249–56.
